# P-15. Impact of Infectious Disease Consultation in Outcome of Enterococcal Bloodstream Infections

**DOI:** 10.1093/ofid/ofaf695.246

**Published:** 2026-01-11

**Authors:** Deepak Kumar, Tejasvi kanagiri, Navneet Kaur, Naresh Kumar Midha, Durga Shankar Meena, sadik mohammed, Vibhor Tak, Gopal Krishna Bohra

**Affiliations:** All India Institute of Medical Sciences, Jodhpur India, Jodhpur, Rajasthan, India; AIIMS, Jodhpur, Jodhpur, Rajasthan, India; All India Institute of Medical Sciences, Jodhpur, Jodhpur, Rajasthan, India; All India Institute of Medical Sciences, Jodhpur, Rajasthan, India; ALL INDIA INSTITUTE OF MEDICAL SCIENCES, JODHPUR, Jodhpur, Rajasthan, India; ALL INDIA INSTITUTE OF MEDICAL SCIENCES,JODHPUR, jodhpur, Rajasthan, India; AIIMS Jodhpur , Jodhpur, Rajasthan, India; All India Institute of medical sciences, Jodhpur, Rajasthan, India

## Abstract

**Background:**

Enterococcus spp is the leading cause of Blood Stream Infections (BSI), particularly in healthcare settings associated with high (20%) mortality (1). Risk factors, co-morbidities, endocarditis, presence of complicated bacteraemia, antibiotic resistance, and inappropriate antibiotic choice of therapy further complicate the E BSI. Timely Infectious Diseases (ID) consultation may improve outcomes through optimized antimicrobial use, source identification, and infection control. (2)(3). This study evaluated the impact of ID consultation on outcomes in patients with EBSI.

**Table 1:**
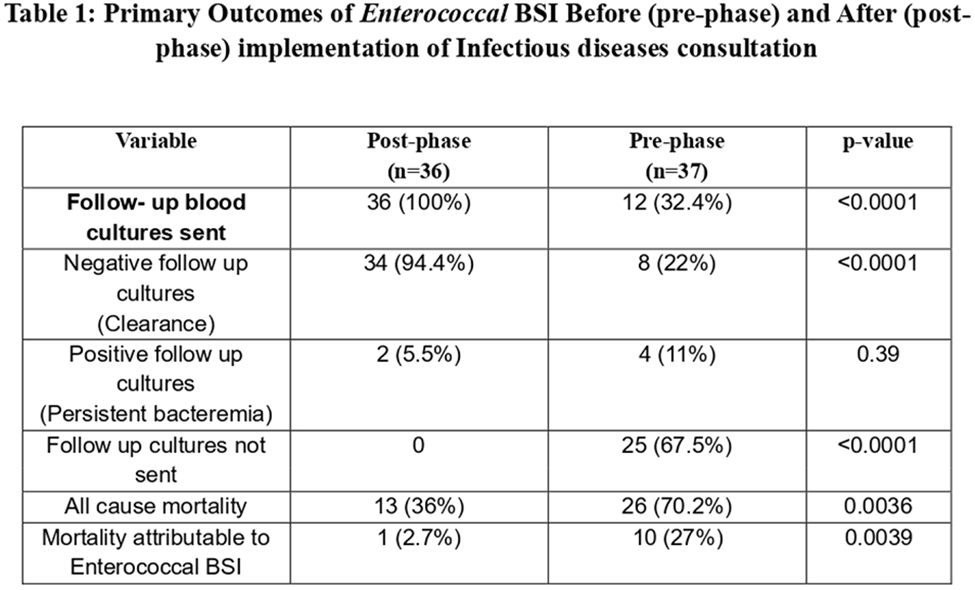
Primary Outcomes of Enterococcal BSI Before (pre-phase) and After (post-phase) implementation of Infectious diseases consultation

**Table 2:**
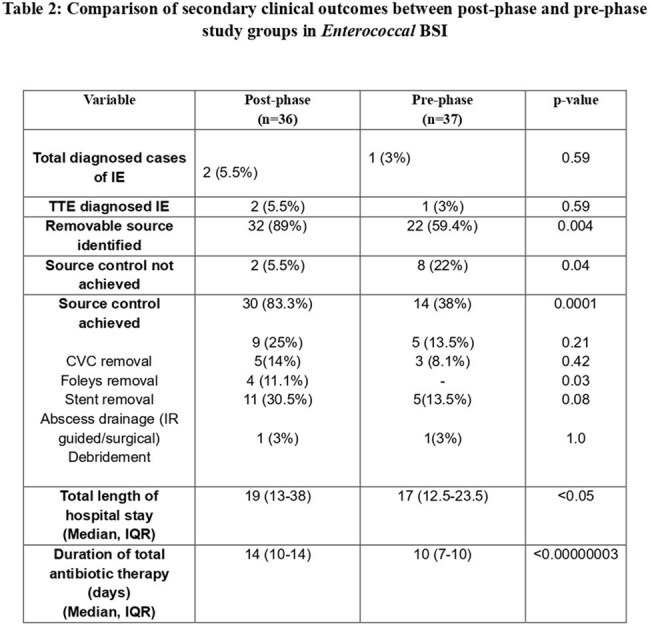
Comparison of secondary clinical outcomes between post-phase and pre-phase study groups in Enterococcal BSI

**Methods:**

A single-center, pre-post interventional study was conducted among hospitalized patients with first episodes of EBSI. In the pre-intervention phase (Jan 2022–June 2023), patients were retrospectively included. In the post-intervention phase (July 2023–Dec 2024) patients were prospectively enrolled and received ID consultation within 24 hours of blood culture positivity. Primary outcomes were bacteremia clearance within 72 hours and all-cause 30-day mortality. Secondary outcomes included source control, endocarditis diagnosis, antibiotic duration, and hospital stay.

**Figure:**
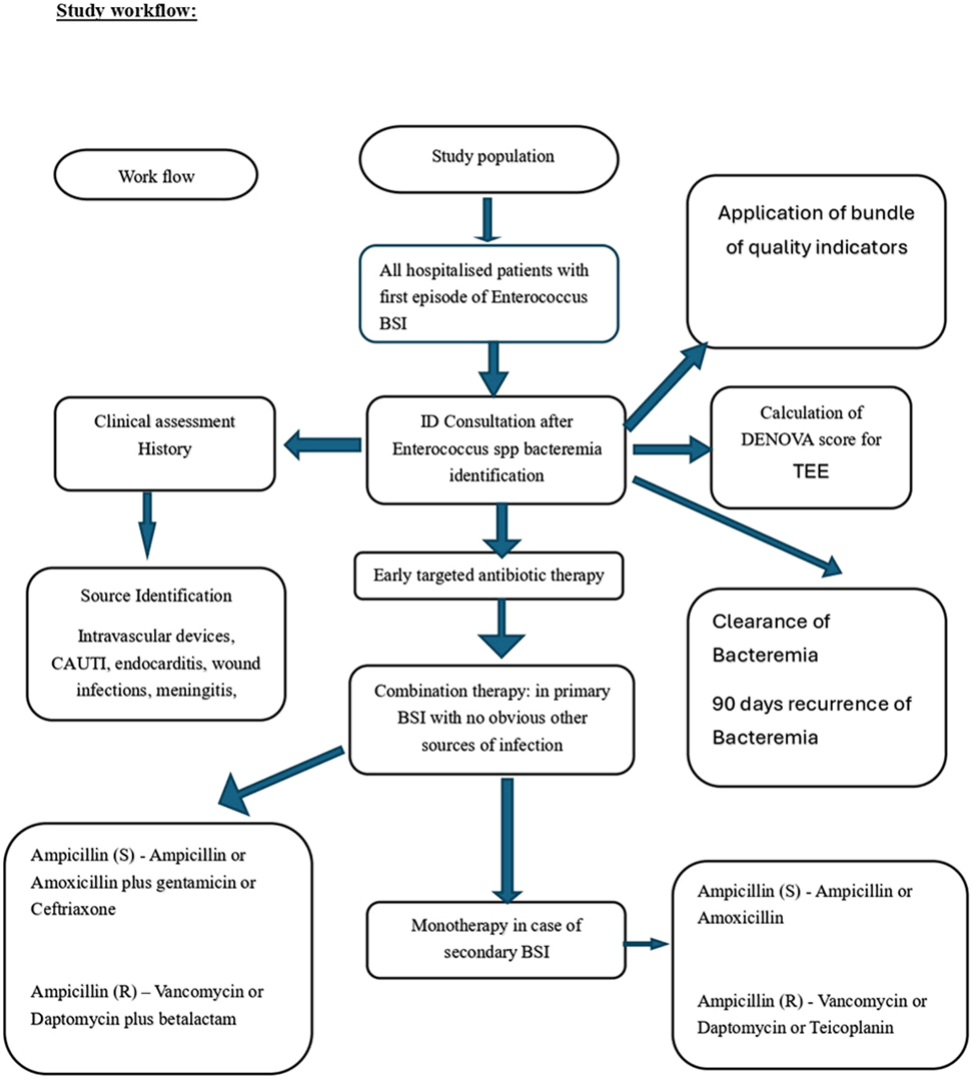
Study work flow

**Results:**

A total of 73 patients with EBSI were included with 36 in the pre-phase phase and 37 in the post-phase.Follow-up blood cultures were obtained in 100% of post-phase patients versus 32.4% pre-phase (p < 0.0001), with higher bacteremia clearance (94.4% vs 22%, p < 0.0001) and lower all-cause mortality (36% vs 70.2%, p = 0.0036) and attributable mortality (2.7% vs 27%, p = 0.0039) as shown in table 1. Removable source identification (89% vs 59.4%, p = 0.004) and source control (83.3% vs 38%, p = 0.0001) were significantly better post-phase. Interventions such as abscess drainage (30.5% vs 13.5%, p = 0.08) were more common in post-phase group. Hospital stay was slightly longer in the post-phase (19 vs 17 days, p < 0.05) likely reflecting thorough evaluation and management. Median duration of total antibiotic therapy duration was extended in post phase consistent effective management (14 vs 10 days, p < 0.00000003) as shown in table 2.

**Conclusion:**

Early ID consultation significantly improved bacteremia clearance, reduced mortality, and enhanced source identification and control in EBSI

**Disclosures:**

All Authors: No reported disclosures

